# Glenoid component implantation accuracy using a novel handheld computer targeting system in total shoulder arthroplasty: a cadaveric study

**DOI:** 10.1016/j.jseint.2026.101663

**Published:** 2026-02-11

**Authors:** Kevin A. Hao, Riccardo Signoretti, Jennifer L. Gass, Obinna I. Nwanna, Deniz K. Kortan, James R. Sibrel, Olivia S. Ng, Mark E. Rogers, H. Scott Ellsworth, Eugene W. Brabston, Joseph J. King

**Affiliations:** a Department of Orthopaedic Surgery & Sports Medicine, University of Florida, Gainesville, FL, USA; b LinkBio Corp., Dover, NJ, USA; c OrthoAlabama Spine & Sports, Birmingham, AL, USA; d Kansas City Orthopedic Alliance, Leawood, KS, USA; e Department of Orthopaedic Surgery, University of Alabama at Birmingham, Birmingham, AL, USA

**Keywords:** Shoulder replacement, Patient-specific instrumentation, Navigation, Reality, Technology, Baseplate, Malposition

## Abstract

**Background:**

The importance of glenoid component positioning in total shoulder arthroplasty (TSA) is increasingly being recognized. Several technologies have been developed to help surgeons accurately replicate their pre-operative plan; however, adoption of these technologies is limited by cost, implant specificity, lack of intraoperative flexibility, and added operating room time. Herein, we evaluate the accuracy of a novel, implant-agnostic, reusable handheld computer targeting system that assists surgeons in placing a central K-wire during TSA using an *in vitro* cadaveric model.

**Methods:**

A cadaveric study was performed wherein 11 matched pairs of fresh-frozen upper torsos (22 shoulders) underwent either anatomic or reverse TSA by 1 of 4 fellowship-trained surgeons. After pre-operative planning and a standard deltopectoral approach, surgeons registered the Computerized Operating Room Experience (CORE) Shoulder system (LinkBio Corp, NJ, USA) to the patients' glenoid. Surgeons then used the wireless handheld device to place the K-wire guided by on-screen targeting cues. Custom anatomic and reverse TSA glenoid test components with metal trackers were subsequently implanted with the retained K-wire, and specimens underwent computed tomography scanning to enable digital comparison to the pre-operative plan. Per convention, a component was considered malpositioned when version or inclination errors exceeded 10° or component displacement exceeded 4 mm. Time from the start of CORE Shoulder glenoid registration to K-wire drilling was calculated from video recordings.

**Results:**

K-wires were accurately positioned in 91% of cases. The mean displacement from the pre-operative plan was 2.1 ± 1.1 mm, and the mean version and inclination errors were 2.2 ± 1.4° and 4.3 ± 4.1°, respectively. Glenoid components were accurately positioned in 95% of cases. The mean displacement from the pre-operative plan was 1.9 ± 1.0 mm, and the mean version and inclination errors were 2.5 ± 1.9° and 3.9 ± 3.1°, respectively. The mean intraoperative time from registration to start of K-wire drilling was 79 ± 24 seconds (range, 44 seconds to 126 seconds). In 95% of cases, registration of the CORE Shoulder device and drilling took less than 2 minutes.

**Conclusion:**

Use of a novel, implant-agnostic, reusable handheld computer targeting system to guide K-wire drilling during *in vitro* anatomic and reverse TSA enabled surgeons to accurately replicate their pre-operative plan in 95% of cases. The use of the CORE Shoulder system added an average of 79 seconds to the procedure (<2 minutes in 95% of cases), supporting its promise in improving surgeon accuracy with minimal added cost and preserved operating room efficiency.

Anatomic and reverse total shoulder arthroplasty (TSA) are very difficult procedures due to challenging surgical exposure, lack of orienting structures on the scapula, and variability in posterior and superior erosive changes. As surgical techniques have progressed and patient outcomes have been scrutinized, a growing body of evidence has developed over the last 2 decades to guide component implantation targets. Although exceptions exist, there is general agreement that anatomic TSA glenoid components should be implanted with >80-90% backside contact,^26^ <10-15° of retroversion,^12^ and in <10° of superior inclination.^28^ Similarly, most surgeons believe that reverse TSA baseplates should be implanted in neutral or inferior inclination,^8^^,^^21^ <10° of retroversion,^5^ and some combination of lateralization and distalization to avoid scapular impingement and excessive soft tissue tension.^1^

Although parameters for implantation have been defined and the advent of pre-operative planning has enabled surgeons to anticipate which implants will optimize biomechanics,^14^ surgeons' ability to execute their pre-operative plan remains challenging due to limited visualization and variable glenoid morphology. In fact, one clinical study wherein experienced fellowship-trained shoulder surgeons simulated placement of a central-axis guide pin in the glenoid with standard instrumentation alone during shoulder arthroplasty found that 38% of cases would be considered malpositioned (>4 mm of displacement or >10° error in version or inclination).^20^ Rates of malposition are especially high for posteriorly worn glenoids,^9^ which are also at the highest risk of clinical and radiographic failure.

In recognition of the challenges associated with glenoid component implantation, there has been increasing interest from surgeons and industry to develop assistive technologies to improve implant placement. This includes the use of patient-specific instrumentation (PSI) using custom-made guides, computer navigation, augmented reality, and robotics.^10^^,^^11^ Although there is consensus that these technologies result in more accurate placement of implants,^24^ widespread adoption of many of the currently available systems for instrumented glenoid component implantation is limited by increased operating room time, availability, cost, added case complexity, and lack of robust demonstration of improved patient outcomes in the literature.

The purpose of this study was to use a cadaveric model to evaluate the accuracy of a simple, implant-agnostic reusable handheld computer targeting system designed to facilitate accurate K-wire placement during TSA. We hypothesized that this system would facilitate efficient and accurate glenoid component implantation during TSA.

## Materials and methods

A cadaveric study was conducted at a single institution's surgical skills laboratory (University of Florida, Gainesville, FL, USA) in September of 2023 to evaluate the accuracy of a novel computer targeting system in accurately placing a central Kirschner (K) wire (guide pin) into the glenoid *in vitro* by representative surgeon users.

### Surgeons

Participants included 4 fellowship-trained surgeons in shoulder and elbow surgery and/or sports medicine with prior experience using a form of enhanced instrumentation, either in the form of PSI guides or computer navigation (Table I). They represented varying levels of experience, and none had used the targeting system used in this study in the clinical setting.

**Table I:** Surgeon user characteristics reported at the time of screening.

User	Years of experience∗	Fellowship	System most used
Surgeon 1	7 yr	Sports medicine	PSI or standard instruments
Surgeon 2	9 yr	Shoulder, elbow, and sports medicine	PSI or standard instruments
Surgeon 3	11 yr	Shoulder and elbow surgery	Computer navigation or standard instruments
Surgeon 4	4 yr	Shoulder and elbow surgery	Arthrex VIP with 5D guide or standard instruments

*PSI*, patient-specific instrumentation; *VIP,* virtual implant positioning.

∗ At the time of study participation.

### Specimens

This cadaveric study included 11 fresh-frozen upper-torso specimens with intact arms, resulting in 11 matched pairs of shoulders (Table II). Three surgeons (surgeons 1, 3, and 4) reviewed pre-operative computed tomography (CT) scans of each shoulder and classified it based on the modified Walch classification. In 50% of specimens (11/22), the classification was unanimous. In 50% of specimens (11/22), there was 1 dissenting classification; thus, the majority classification was adopted. There were no specimens for which all 3 surgeons had conflicting classifications. Specimens were randomized to receive either anatomic or reverse TSA; 7 left-sided shoulders were designated to undergo anatomic TSA, and 4 to undergo reverse TSA. Consequently, 4 right-sided shoulders received an anatomic TSA, and 7 received a reverse TSA.

**Table II:** Characteristics of included cadaveric specimens.

Specimen #	Laterality	Implant	Walch classification	Surgeon	Guide size (mm)	Native version	Native inclination	AP diameter (mm)	SI diameter (mm)	Planned version	Planned inclination
1	Left	Anatomic	A1	Surgeon 2	24 mm	8°	23°	29	37	0°	27°
2	Right	Reverse	A1	Surgeon 2	20 mm	−6°	9°	26	37	−5°	0°
3	Left	Anatomic	B1	Surgeon 1	24 mm	−15°	8°	26	39	−7°	12°
4	Right	Reverse	B1	Surgeon 1	24 mm	−16°	1°	31	40	−8°	0°
5	Left	Anatomic	A2	Surgeon 1	28 mm	−12°	30°	31	47	−12°	37°
6	Right	Reverse	A2	Surgeon 1	24 mm	2°	19°	27	42	0°	10°
7	Left	Anatomic	A1	Surgeon 2	20 mm	−7°	12°	27	38	−6°	0°
8	Right	Reverse	B3	Surgeon 2	20 mm	−18°	4°	32	45	−10°	0°
9	Left	Anatomic	B1	Surgeon 1	28 mm	0°	17°	32	43	0°	7°
10	Right	Reverse	B1	Surgeon 1	28 mm	−3°	16°	33	41	0°	7°
11	Left	Anatomic	A1	Surgeon 4	24 mm	−2°	8°	29	41	0°	5°
12	Right	Reverse	A2	Surgeon 4	24 mm	−4°	5°	28	43	0°	0°
13	Left	Anatomic	A1	Surgeon 3	16 mm	−12°	21°	20	33	0°	10°
14	Right	Reverse	A1	Surgeon 3	20 mm	−3°	17°	18	29	0°	10°
15	Left	Anatomic	A1	Surgeon 3	20 mm	−3°	14°	21	32	0°	0°
16	Right	Reverse	B1	Surgeon 3	20 mm	−6°	13°	20	31	0°	8°
17	Left	Anatomic	A2	Surgeon 2	20 mm	−19°	28°	27	43	0°	10°
18	Right	Reverse	A2	Surgeon 2	20 mm	−3°	−4°	27	40	0°	0°
19	Left	Anatomic	A1	Surgeon 4	24 mm	2°	11°	27	43	0°	5°
20	Right	Reverse	B1	Surgeon 3	24 mm	−1°	17°	29	42	0°	0°
21	Left	Anatomic	A1	Surgeon 4	24 mm	5°	11°	24	41	0°	5°
22	Right	Reverse	A1	Surgeon 4	24 mm	3°	6°	28	50	0°	0°

*AP*, anteroposterior; *SI*, superoinferior.

### Targeting system

**Figure 1:**
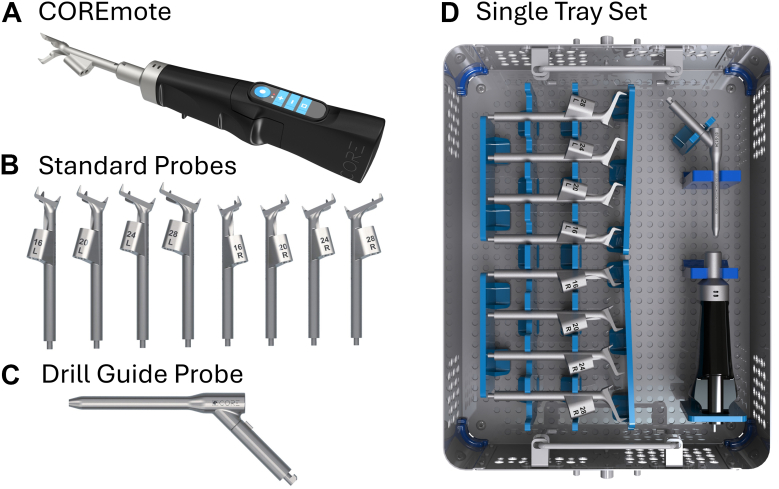
CORE Shoulder sterile field instrumentation. CORE Shoulder instrumentation consists of the COREmote (**A**), standard probe set consisting of 4 left-sided and 4 right-sided guides (**B**), and the optional drill guide probe (**C**). All sterile field reusable components fit inside a single tray (**D**). *CORE*, Computerized Operating Room Experience.

The Computerized Operating Room Experience (CORE) Shoulder system (LinkBio Corp, NJ, USA) is a handheld surgical instrument that assists surgeons with accurate placement of a K-wire into the glenoid during primary TSA. The system consists of a handheld remote (COREmote; Fig. 1*A*), which includes a sensor unit and power unit, as well as the CORE Workstation, which provides visual instructions and feedback to the surgeon intraoperatively. The workstation communicates with the remote using wireless technology, so it does not require a direct line of sight with the remote. The system guides the placement of a central K-wire, which then guides glenoid reaming and implant placement. Thus, the CORE Shoulder system is implant agnostic and provides implant positioning assistance without any dependency on implant type.

The system offers both a standard probe (Fig. 1*B*) and a drill guide probe (Fig. 1*C*) for final K-wire drilling. The standard probe comes in 16 mm, 20 mm, 24 mm, and 28 mm sizes mirrored for laterality (total of 8 probes). In practice, these probes are interchangeable, and surgeons may elect to use the angled drill guide probe based on retractor interference and/or to obtain a better view of the glenoid. All reusable sterile components of the CORE Shoulder system fit in a single tray (Fig. 1*D*). As the focus of this study was to evaluate the accuracy of the CORE Shoulder system using the standard probe, 2 shoulders were randomly designated for use of the drill guide probe for the trajectory targeting step, whereas the remaining 20 shoulders were instrumented with the standard probe for the whole workflow.

### Test implants

The primary outcome of this study was the deviation of K-wire placement relative to the pre-operative plan. Therefore, it was necessary to retain K-wires in the cadaveric specimens for CT scan. Thus, in order to measure implantation accuracy of both the K-wire and implant simultaneously, test implants were manufactured with a center hole for the ⌀ 2.4 mm K-wire to pass through. Test implants that were geometrically equivalent to the anatomic and reverse TSA implants in the LINK Embrace system (Waldemar Link GmbH & Co. KG, Hamburg, Germany) were 3-dimensional (3D)-printed of HP Multi Jet Fusion PA12 (Polyamide) and embedded with radioopaque spheres to enable detection on post-operative CT scans. The surgical technique for implanting the glenoid test implants was similar to the Embrace system.

### Study protocol

CT scans of the cadaveric specimens were obtained, and pre-operative planning was performed in mediCAD 3D Shoulder (mediCAD Hectec GmbH, Altdorf, Germany) by an employee (M.A.) experienced with TSA. Implants were planned in varying positions to represent a range of potential plans, and the pre-operative plan was not necessarily intended to represent a clinical plan. Prior to this study, the plan was reviewed and approved by the operating surgeon. If desired, surgeons were able to revise the pre-operative plan.

**Figure 2:**
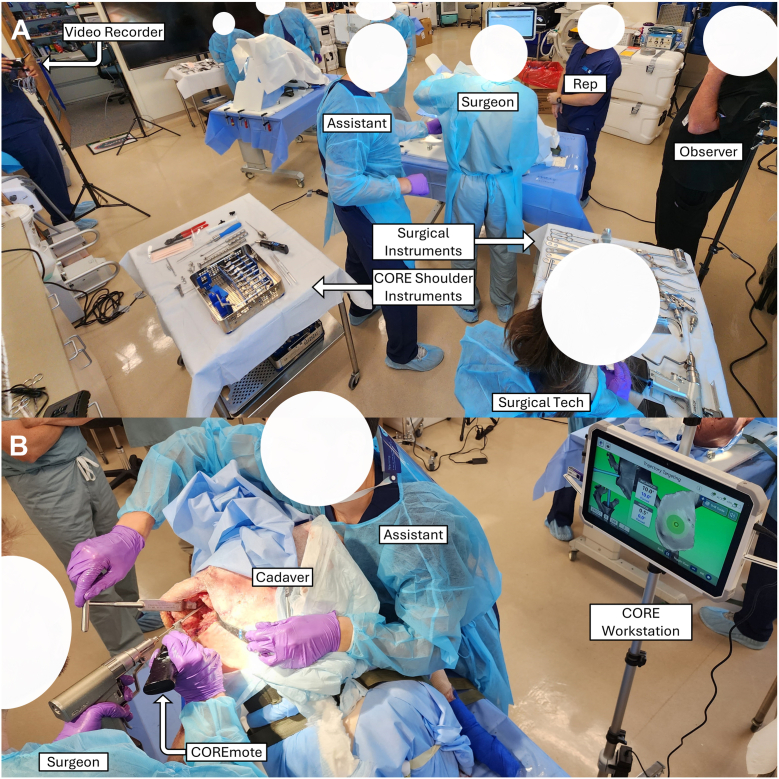
Representative pictures of surgeon 2's test setup (**A**) and targeted K-wire drilling (**B**).

**Figure 3:**
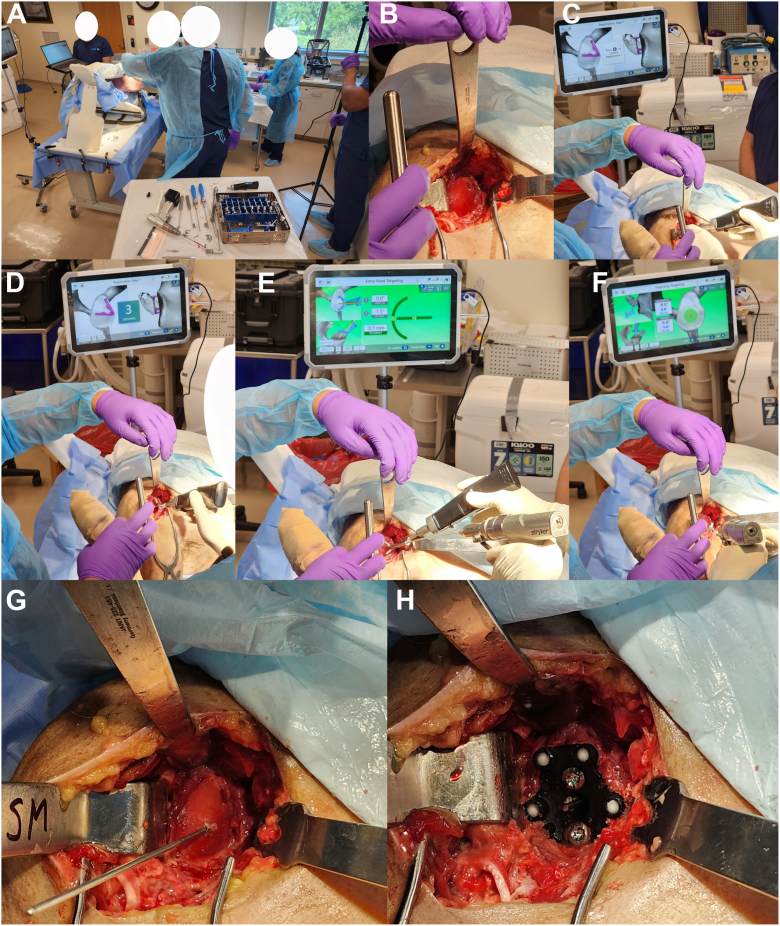
Representative case of reverse TSA implanted with the CORE Shoulder system. Room setup consists of the CORE workstation, right-sided upper-torso, video camera, and back table with CORE instrumentation (**A**). After a standard deltopectoral approach with glenoid exposure and preparation (**B**), the COREmote is registered to the glenoid by aligning the spikes of the probe with the appropriate anatomic landmarks (**C**) and holding the position for 3 s (**D**). Next, pivoting on the anterior spike, the COREmote is swiveled to register the second position in the glenoid (not pictured). Pivoting off of the anterior spike, the entry point is identified and marked (**E**). Pivoting off of the K-wire, the probe can be retracted along the K-wire and appropriate version and inclination can be determined (**F**). The K-wire is then advanced bicortically, completing the targeting phase of the procedure (**G**). Guided by the K-wire, standard reverse TSA technique is used to ream the glenoid and implant a custom plastic baseplate with metal trackers (**H**). *CORE*, Computerized Operating Room Experience; *TSA*, total shoulder arthroplasty.

**Figure 4:**
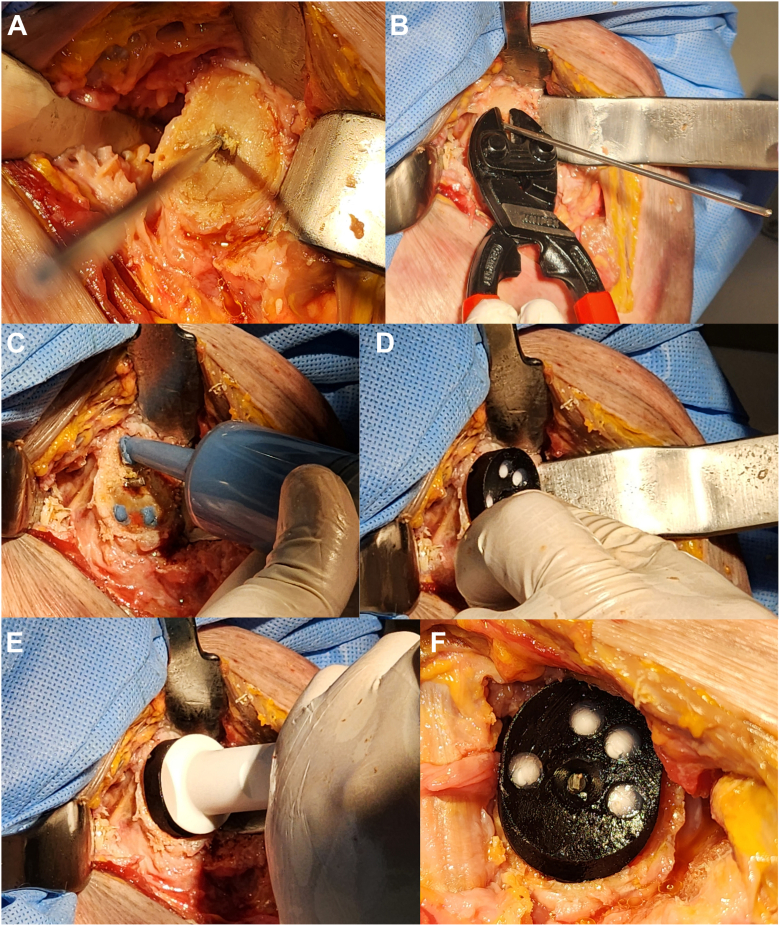
Representative case of anatomic TSA after K-wire implantation. After placement of the K-wire using the CORE Shoulder system and glenoid reaming, standard anatomic TSA surgical technique is used to ream the glenoid and drill peg holes (**A**). The K-wire is clipped (**B**), peg holes are cemented (**C**), and the custom glenoid component with metal trackers is implanted (**D**) and impacted (**E**) into the glenoid. The hemi upper-torso with both the K-wire and custom glenoid component is shown (**F**). *CORE*, Computerized Operating Room Experience; *TSA*, total shoulder arthroplasty.

Cadaveric specimens were mounted in a beach chair position (Fig. 2). Surgeons performed a standard deltopectoral approach, humeral head cut, labrum excision, and cartilage removal. During the pre-operative setup, the COREmote was assembled by connecting the power and sensor units, connected wirelessly to the workstation computer graphical user interface (GUI), assembled with the probe, and placed on a flat table for calibration. The COREmote was then registered to the glenoid by aligning the three-pronged probe such that its spikes contacted the glenoid based on the first set of pre-operatively planned registration points. Pivoting on the anterior spike of the probe, the COREmote is rotated and aligned to the second set of registration points, completing the registration phase. To confirm accurate registration, the COREmote can be pivoted on the glenoid face by the operating surgeon to verify that the landmarks in the cadaver align to the GUI. After satisfactory registration is confirmed, the COREmote is pivoted on the anterior point, and the workstation GUI is referenced to align the probe according to the pre-operative plan, and a K-wire is drilled through the guide hole of the probe. Alternatively, if switching to the drill guide probe, the entry point is first marked while pivoting with the probe, and then the standard probe can be exchanged with the drill guide probe. After the entry point has been identified, the COREmote no longer needs to contact the glenoid and can be slid back proximally along the K-wire to assist with version and inclination. The surgeon then drills the K-wire bicortically, confirms the placement of the K-wire on the computer GUI, and then the COREmote is slid off of the K-wire, completing the targeting phase of the procedure. K-wires were retained in the specimens in order to measure their placement accuracy on post-implantation CT scans. After K-wire placement, surgeons used the Link Embrace shoulder system and surgical technique to ream the glenoid and implant the glenoid test component (either anatomic or reverse TSA). Representative images of the workflow are provided in Figs. 3 and 4.

### Study outcomes

The primary outcome of this study was the accuracy of K-wire placement relative to the surgeon's pre-operative plan. Secondary outcomes included accuracy of glenoid component placement relative to the pre-operative plan and intraoperative time added by use of the CORE Shoulder system. Simulated intraoperative times were calculated retrospectively upon reviewing video footage of each procedure. Surgeons were asked to perform the operation at their standard pace and were blinded to the fact that operative time would be evaluated. To measure errors between pre-operative plans and post-operative K-wire and glenoid component placement, all specimens underwent a post-operative CT scan to capture the implant and K-wire positions in relation to the native scapula. 3D Slicer (version 5.4.0)^6^ was used to segment the components of the post-operative scan, then MeshLab (version 2022.02, CNR-ISTI Visual Computing Lab, Pisa, Italy) was used to align the post-operative scan with the pre-operative scan. Using Blender (version 3.5, Blender Foundation, Amsterdam, Netherlands), the positions and orientations of the implant and K-wire were calculated. Subsequently, the post-operative K-wire and implant were compared against the pre-operative planned implant position. Component displacement in the sagittal plane was measured in the anteroposterior (AP) and superoinferior (SI) axes, and a vectorized displacement (combined offset) was calculated. Version and inclination errors were calculated relative to the axis of the pre-operative plan.

Each case was filmed in its entirety and reviewed post-operatively. Using visual and auditory cues in the video, we recorded the intraoperative time that it took the user to complete the CORE workflow. Intraoperative time was defined as the time between the start of registration and the start of K-wire insertion. In cases where the surgeon started the workflow, but stopped or paused to restart the registration process, the start time of the later registration was used. This allowed for direct comparison between CORE procedure times across cases. Reasons for restarting the registration process were concern that the probe position relative to the glenoid was inaccurate after initial registration or the probe slipped on the face of the glenoid. Surgeons made the decision autonomously to restart the registration process.

### Statistical analysis

We adopted a definition of component malpositioning based on previously published criteria: version or inclination errors exceeding 10° or starting point displacement exceeding 4 mm.^9^^,^^20^^,^^22^ Descriptive statistics were calculated to estimate the incidence of malpositioning of the K-wire and subsequently implanted glenoid component compared to the pre-operative plan. All positional and angular errors were expressed as absolute values when aggregating descriptive statistics. Vectorized errors (+/−) were portrayed in figures to evaluate trends. Analytics were performed in R Software (version 4.2.0, R Core Team, Vienna, Austria).

## Results

The 11 matched pairs of cadaveric upper torsos (22 shoulders) were included. The mean retroversion was 5 ± 8° (range, 19° of retroversion to 8° of anteversion), and the mean inclination was 13 ± 8° (range, 4° of inferior inclination to 30° of superior inclination). The mean AP width of the glenoid was 27 ± 4 mm, and the mean SI length of the glenoid was 40 ± 5 mm. The Walch classification of the sample included 10 A1 (45%), 5 A2 (23%), 6 B1 (27%), 0 B2 (0%), and 1 B3 (5%). The mean intraoperative time from registration to start of K-wire drilling was 79 ± 24 seconds (range, 50 seconds to 126 seconds). In 95% of cases, registration of the CORE Shoulder device and preparation for targeted placement of the K-wire took less than 2 minutes. In 6 cases (27%), the surgeon restarted the registration process; in these cases, an additional 81 ± 62 seconds (range, 28 to 170 seconds) was added. When accounting for all registration attempts, registration of the CORE Shoulder device and preparation for targeted placement of the K-wire took less than 2 minutes in 77% of cases and less than 3 minutes in 91% of cases.

### K-wire accuracy

**Figure 5:**
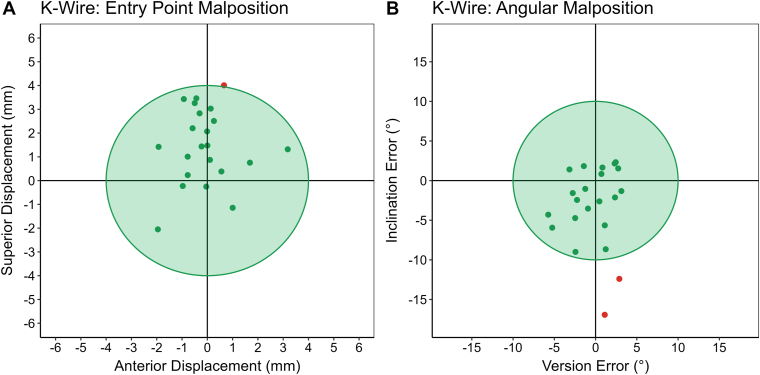
Entry point (**A**) and angular malposition (**B**) of K-wires placed with the CORE Shoulder device relative to the pre-operative plan. K-wires met all acceptable placement criteria (≤4 mm displacement, ≤10° version error, and ≤10° inclination error) in 91% of cases. *CORE*, Computerized Operating Room Experience.

K-wires met all acceptable placement criteria (≤4 mm displacement, ≤10° version error, and ≤10° inclination error) in 91% of cases (Fig. 5). The mean AP and SI offset error was 0.8 ± 0.8 mm and 1.8 ± 1.2 mm, with a vectorized mean offset of 2.1 ± 1.1 mm (Table III). The mean version and inclination error were 2.2 ± 1.4° and 4.3 ± 4.1°, respectively. In general, there was a slight tendency to place the K-wire too superior on the glenoid with too much inferior inclination relative to the pre-operative plan.

**Table III:** Summary statistics of K-wire and glenoid component placement deviance from the pre-operative plan validated on post-operative CT scans.

Measurement	Mean ± SD	Median [IQR]	Range
K-wire			
AP offset error (mm)	0.8 ± 0.8	0.6 [0.2-1.0]	0.0-3.2
SI offset error (mm)	1.8 ± 1.2	1.5 [0.9-2.7]	0.2-4.0
Combined offset error (mm)	2.1 ± 1.1	2.2 [1.3-3.0]	0.3-4.1
Version error (°)	2.2 ± 1.4	2.3 [1.1-2.8]	0.5-5.7
Inclination error (°)	4.3 ± 4.1	2.4 [1.6-5.4]	0.8-16.9
Glenoid component			
AP offset error (mm)	0.8 ± 0.8	0.6 [0.2-1.2]	0.0-3.1
SI offset error (mm)	1.5 ± 1.1	1.3 [0.4-2.3]	0.0-3.7
Combined offset error (mm)	1.9 ± 1.0	1.8 [1.4-2.6]	0.3-3.8
Version error (°)	2.5 ± 1.9	1.9 [1.0-3.4]	0.1-6.1
Inclination error (°)	3.9 ± 3.1	2.8 [1.4-5.3]	0.0-11.2

*AP*, anteroposterior; *CT*, computed tomography; *IQR*, interquartile range; *SD*, standard deviation; *SI*, superoinferior.

### Glenoid component accuracy

**Figure 6:**
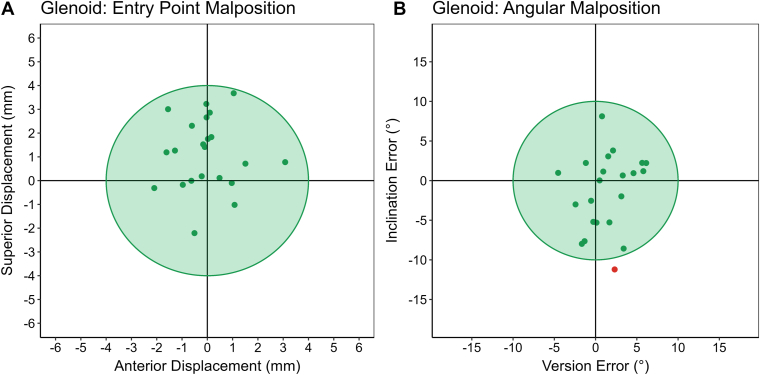
Entry point (**A**) and angular malposition (**B**) of implanted glenoid components placed based on the K-wire drilled using the CORE Shoulder device relative to the pre-operative plan. Implanted glenoid components met all acceptable placement criteria (≤4 mm displacement, ≤10° version error, and ≤10° inclination error) in 95% of cases. *CORE*, Computerized Operating Room Experience.

Glenoid components met all acceptable placement criteria in 95% of cases (Fig. 6). The mean AP and SI offset error was 0.8 ± 0.8 mm and 1.5 ± 1.1 mm, with a vectorized mean offset of 1.9 ± 1.0 mm (Table III). The mean version and inclination error was 2.5 ± 1.9° and 3.9 ± 3.1°, respectively. Similar to the K-wire, there was a slight tendency to place the glenoid component too superior on the glenoid. However, there was no pattern observed with regard to error in version and inclination relative to the pre-operative plan.

## Discussion

In this study, we evaluated the use of a novel, implant-agnostic, reusable handheld computer targeting system to perform K-wire placement during *in vitro* anatomic and reverse TSA. When benchmarked to a prior definition of component malpositioning (≤4 mm displacement, ≤10° version error, and ≤10° inclination error), we found that this handheld targeting system enabled surgeons to place the K-wire and glenoid component accurately according to their pre-operative plan in 91% and 95% of cases, respectively. When added time to the case was evaluated, we found that the use of the CORE Shoulder system (registration and targeting steps) added an average of 79 seconds to the case and less than 2 minutes in 95% of cases.

There is increasingly compelling evidence to suggest targets for implantation of anatomic total shoulder arthroplasty^4^^,^^12^^,^^26^ and reverse total shoulder arthroplasty^8^^,^^18^^,^^21^ components to optimize range of motion, to minimize complications such as scapular notching and instability, and to maximize implant survivorship. Pre-operative planning has allowed surgeons to understand how much correction of the joint line is needed, anticipate intraoperative challenges, and appropriately size implants. However, prior studies have demonstrated that pre-operative planning alone is not sufficient to accurately place components, with 1 study demonstrating a malposition rate of 38% in expert hands despite the use of pre-operative planning.^20^

Numerous other technologies exist aimed at assisting surgeons with reproducing their pre-operative plan, including PSI, computer navigation, augmented reality, and robotics. However, they all have their own limitations. While PSI requires several weeks of lead-time to manufacture and ship the custom guide, the computer navigation systems can be used on the same day as the CT scan. Additionally, the pre-operative plan can be changed intraoperatively with computer navigation, whereas PSI guides cannot be adjusted if they do not fit the glenoid as anticipated. Most computer navigation, augmented reality, and robotics systems utilize a tracker that is attached to the scapula (commonly to the coracoid process), adding time and complexity to the case and introducing the risk of an intraoperative coracoid fracture or tracker loosening.^17^^,^^19^ In contrast to these systems, the CORE system relies on the user maintaining contact between the anterior spike of the probe and the anterior glenoid rim to maintain its position, without movement of the glenoid retractors or the scapula during registration and K-wire drilling. While this drastically improves the efficiency of the system, it also inevitably presents the drawback of having to re-calibrate if the contact between the probe and the glenoid is lost. However, registration of the CORE system is expeditious, as demonstrated in this study with a mean time from registration to K-wire drilling of 79 seconds. While most navigation systems require many points on the scapula to be registered during the calibration process, the CORE system only requires 5 points, contributing to its efficiency. While this may be expected to limit its accuracy compared to navigation systems, the results of the present study suggest the CORE Shoulder targeting system has similar accuracy to other systems reported in the literature.

The accuracy of K-wire and glenoid component placement demonstrated in the present study using the CORE Shoulder system compare favorably to prior literature. Meta-analyses pooling the rate of component malpositioning (using the same definition as the current study) in human and cadaveric studies utilizing PSI have reported a range between 15-20%,^2^^,^^27^ compared to our 9% and 5% rates for K-wire and glenoid component malposition, respectively. In a comparative study of 2 consecutive groups of 28 patients who underwent reverse TSA by a single surgeon with and without navigation (NextAR system, Medacta, Castel San Pietro, Switzerland), Cunningham et al^3^ found that use of navigation resulted in improved replication of the pre-operative plan during reverse TSA when post-operative CT scans were evaluated. In another series comparing replication of the pre-operative plan with and without computer navigation (ExactechGPS, Exactech Inc., Gainesville, FL) in cadavers, Jones et al^15^ found that errors in baseplate positioning during reverse TSA were lower when navigation was used for AP positioning (0.7 mm vs. 1.4 mm; *P* = .060), SI positioning (1.1 mm vs. 1.4 mm; *P* = .457), version (1.9° vs. 5.9°; *P* = .004) and inclination (2.4° vs. 6.3°; *P* = .026). When looking only at reverse TSA baseplate positioning error relative to the pre-operative plan from the current study, errors in AP positioning (0.8 mm), SI positioning (1.6 mm), version (2.8°), and inclination (3.9°) appear similar to this prior study.

While the evidence supporting improved implementation of surgeons' pre-operative plan using technologies such as PSI, navigation, and robotics is strong, evidence of improved clinical outcomes is still pending.^7^^,^^13^^,^^23^^,^^25^ In a minimum 2-year follow-up study, Holzgrefe et al^13^ matched 113 navigated reverse TSAs to non-navigated shoulders based on sex, age, follow-up, and pre-operative diagnosis and demonstrated similar clinical outcomes, but a trend towards a 3.5-times higher revision rate when navigation was not used (3.5% vs. 0.9%). Another study comparing 216 anatomic and 533 reverse TSAs performed with navigation to a matched cohort of patients with standard instrumentation found that navigation during reverse TSA was associated with an absolute risk reduction of 1.7% (95% confidence interval: 0%, 3.4%) for post-operative complications and 0.7% (95% confidence interval: 0.1%, 1.2%) for dislocation.^29^ While some other studies on navigation have found no difference in clinical outcomes with and without navigation, they have identified a lower incidence of vault penetration and use of longer screws, which may improve long-term survivorship of the baseplate.^16^ Future studies with longer-term follow-up are needed to ascertain the impact of improved component placement on the durability of functional outcomes and implant survivorship.

There were 2 shoulders that did not meet acceptable implantation criteria in this study. These were the first 2 cases performed by Surgeon 2. Video recordings were retrospectively reviewed, and it was observed that the surgeon and assistant (holding the retractors) did not follow instructions and deviated from protocol by repositioning retractors during use of the CORE device, leading to progressively worse results. The first attempt was unnoticed by the test proctor, and the K-wire was inferiorly inclined by 12° relative to the pre-operative plan. In the second attempt, the repositioning was more significant, resulting in a 4.1 mm displacement of the entry point and an inferior inclination error of 17°, which allowed the test proctor to observe the technique error and instruct the user and assistant to stop manually repositioning retractors during use of the CORE device. All subsequent cases by Surgeon 2 and their assistants were performed correctly and met accuracy criteria. As a result of this observation, the CORE software has been updated to specifically warn users not to move the retractors while the CORE device is being used to target the K-wire. Furthermore, an additional “K-wire check” step has been added prior to final placement of implants.

Strengths of this study include a generalizable user group comprised of sports medicine and shoulder surgeons who perform TSA, use of upper-torso cadavers with matched pairs undergoing anatomic and reverse TSA, and the use of post-operative CT scans to evaluate accuracy. Additionally, surgeons were blinded to the fact that the time from registration to K-wire insertion would be an outcome of the study, and surgeons chose to re-do the registration on more than one occasion when they were concerned that the device had shifted or they did not find the GUI representation of the probe on the glenoid to be accurate; despite this, 95% of K-wire targeting cases were performed in under 2 minutes. In addition to K-wire positioning, glenoid component implantation was also simulated, and this study demonstrated similar deviations from the pre-operative plan for both the K-wire and glenoid components, further supporting the validity of this technique.

There are several limitations of this study. First, it was performed *in vitro*, removing many of the challenges of TSA encountered *in vivo* such as suboptimal exposure and bleeding. Second, a relatively low sample size at 11 matched pairs of upper torsos was evaluated; a larger sample would improve confidence in our accuracy rates. We did not include a control group to evaluate whether the CORE device improves accuracy over standard instrumentation; however, it has already been well-established that use of standard instrumentation alone, even in expert hands results in frequent malpositioning.^20^ Another limitation is the inclusion of 4 fellowship-trained surgeons who were previously familiar with using technology beyond standard instrumentation in TSA; surgeons who are naïve to using technology during TSA may have a steeper learning curve. Similarly, all 4 surgeons included had limited experience using the CORE Shoulder system, and none had used it intraoperatively; thus, the results of this study likely represent part of their learning curve. Certainly, the lack of clinical outcomes is a limitation of this study, and future studies will be needed to ascertain the role of navigation and targeting on TSA outcomes.

## Conclusion

In this study, we demonstrated that the use of a novel, implant-agnostic, reusable handheld computer targeting system to guide K-wire drilling during *in vitro* anatomic and reverse TSA enabled surgeons to accurately replicate their pre-operative plan in 95% of cases. The use of the CORE Shoulder system took an average of 79 seconds to execute and less than 2 minutes in 95% of cases. Our findings support handheld targeting's promise in improving surgeon accuracy with minimal added cost and preserved operating room efficiency. However, future clinical studies are needed to evaluate how these findings translate to clinical practice.

## Disclaimers:

Funding: No funding was disclosed by the authors.

Conflicts of interest: Mark E. Rogers is a paid consultant for LinkBio Corp. H. Scott Ellsworth is a paid consultant for LinkBio Corp. Eugene W Brabston is a paid consultant for LinkBio Corp. Joseph J. King is a paid consultant for Exactech, Inc. and LinkBio Corp. The other authors, their immediate families, and any research foundations with which they are affiliated have not received any financial payments or other benefits from any commercial entity related to the subject of this article.
